# Comparison of lifestyle in fertile and infertile couples in Kermanshah during 2013

**Published:** 2015-09

**Authors:** Tahereh Khosrorad, Mahrokh Dolatian, Hedyeh Riazi, Zohreh Mahmoodi, Hamid Alavimajd, Soodeh Shahsavari, Mitra Bakhtiari

**Affiliations:** 1 *Department of Midwifery, Shahid Beheshti University of Medical Sciences, Student Research Committee, Tehran, Iran.*; 2 *Department of Midwifery, Shahid Beheshti University of Medical Sciences, Tehran, Iran.*; 3 *Social Determinant of Health Research Center, Alborz Univversity of Medical Sciences, Karaj, Iran.*; 4 *Department of Midwifery, Faculty of Nursing and Midwifery, Alborz University of Medical Sciences, Karaj, Iran.*; 5 *Department of Biostatistics, School of Paramedical Sciences, Shahid Beheshti University of Medical Sciences,Tehran, Iran.*; 6 *Kermanshah University of Medical Sciences, Kermanshah, Iran.*

**Keywords:** *Lifestyle*, *Fertile couples*, *Infertile couples*, *Social support*, *Nutrition*, *Physical activity*, *Health behaviors*

## Abstract

**Background:**

Infertility is a major reproductive health in gynecology. According to the world health organization, there are currently 50-80 million infertile couples in the world.

**Objective:**

Considering the critical effects of lifestyle on reproductive health, this study aimed to compare the lifestyle of fertile and infertile couples in Kermanshah during 2013.

**Materials and Methods:**

This research is a descriptive cross sectional study that was done on 216 fertile and infertile couples attending Infertility Center and six medical centers that were selected through the convenience sampling. Data were collected using a researcher-made questionnaire containing demographic and fertility-related information and also lifestyle items on nutrition, physical activity, perceived social support, responsibility for health, and inappropriate health behaviors. Descriptive statistics, logistic regression analysis, independent t, chi-square and Generalized Estimating equation were performed to analyze the data.

**Results:**

Fertile and infertile women (86.1% and 73. 1% respectively, p= 0. 03) as well as fertile and infertile men were significantly different in terms of physical activity (87% and 96.3% p<0.001, respectively) and perceived social support (p<0.001). Moreover, there was a significant difference between fertile and infertile women in nutrition (p<0.001). Similar differences were observed in responsibility for health and inappropriate health behaviors between fertile and infertile men. However, all of the dimensions of lifestyle, except nutrition, were significantly different between fertile and infertile couples.

**Conclusion:**

As lifestyle plays a crucial role in reproductive health, the inappropriate lifestyle of infertile couples has to be modified through effective measures such as awareness promotion, behavioral changes, and development of a healthy environment.

## Introduction

Infertility is a major reproductive health problem affecting a huge proportion of the young population worldwide. It is experienced by about 10% - 15% of couples early after marriage (1) and by almost one out of seven couples overall (2). The world health organization (WHO) defines infertility as a disease of the reproductive system due to which a non-contraception couple fails to achieve pregnancy over at least 12 months (3). However, other references have sometimes described infertility as failure to achieve pregnancy after two years of unprotected sex (4). The numerous factors leading to infertility in a couple can be attributed to the husband (30%), the wife (40%), or both (39%) (5). Meanwhile, the cause of infertility is still unknown in 30% of the couples. The most frequent causes of infertility in Asian countries, including Iran, are sperm abnormalities, fallopian tube disorders, and ovulatory dysfunction (6).

As the emergence of assisted reproductive technology (ART) has eliminated many problems faced by infertile couples, more couples in developed countries tend to use the technology every day. Needless to say, while this increased demand has imposed great economic burden on public health sector of the mentioned countries, the success rate of the ART is as low as 37% in women under 35 years old, 16% in 40-year old women, and 2% in women over 43 years old (7). Consequently, lifestyle has received more attention as a major factor contributing to general and reproductive health (8). Lifestyle was first introduced as a determinant of health in 1970 and this relationship has been further examined ever since. The WHO has defined healthy lifestyle as a series of behaviors ensuring an individual’s physical and mental health(9). While physical quality of life encompasses nutrition, physical activity, and sleep, mental quality of life deals with social relationships, coping with stress, enquiry and learning, and spirituality (9).

Lifestyle is defined based on certain behavioral patterns produced by the interactions between personal characteristics, social relationships, environmental conditions, and socioeconomic situations (10). MacDonald and Thompson (2005) introduced the dimensions of lifestyle as nutrition, exercising, self-care, smoking, consumption of alcohol and illegal drugs, social relations, and control of stress (11).

Contemporary life has increased specific lifestyle-related factors. For instance, obesity, which may arise from immobility or inappropriate diet, can in turn cause hormone imbalance (8). According to previous researches, improved insulin sensitivity in women with polycystic ovary syndrome can suggest the effects of physical activity on the reproductive system (12). Rovonta et al. (2010) found unsaturated and saturated fat consumption to be respectively 23% and 7% higher in infertile women under 50 years old in fertile women of the same age. The rates were also higher in infertile men (compared to fertile men) by 6%. However, the two groups of women were not significantly different in the level of physical activity (13). Braga et al. (2011) found sperm concentration to be negatively correlated with alcohol use and body mass index and positively correlated with the number of daily meals and the amount of consumed cereals. In addition, sperm motility had negative correlations with not only body mass index, but also alcohol and cigarette use. A positive correlation was also observed between normal sperm morphology and fruit and vegetable intake (14). Twigt *et al*. (2012) proposed diet modification as a method to boost the chance of pregnancy in women undergoing ART (15). Despite the crucial role of lifestyle, as a multifactorial concept based on Macdonald definition, in fertility, it has been rarely studied in Iranian infertile couples. Moreover, the few available studies in this field have merely focused on certain dimensions of this phenomenon. Therefore, the present research attempted to compare the lifestyles of fertile and infertile couples in Iran.

## Materials and methods

The study population in this descriptive analytical study comprised all fertile couples visiting various medical centers in Kermanshah (Iran) and all infertile couples attending Motazedi Infertility Center (Kermanshah, Iran) in 2013 from April to January. The couples were only recruited if they were Iranian, 18-45 years old, and literate. Couples were considered fertile if the woman had given birth to at least one child (over six months before the study) and had gotten pregnant without ART. On the other hand, couples who had an infertility diagnosis for more than a year were regarded as infertile. Couples with a history of chronic diseases and an ongoing pregnancy were not included. While the fertile couples were selected from the six districts of Kermanshah using stratified sampling, convenience sampling was applied to select infertile couples. Finally, the eligible couples who provided written consent entered the study.

Based on statistical calculations, the sample size was determined as 108 fertile and 108 infertile couples. Data were collected through a demographic and fertility/infertility questionnaire containing items on age, ethnicity, occupation, occupational exposure to particular substances, education level, type of ownership, family size, number of pregnancies, and duration and cause of infertility. A lifestyle questionnaire was also prepared to assess the participants’ nutrition, physical activity, inappropriate health behaviors, perceived social support, and responsibility for health. A total of 16 researcher-made items (scored based on a four-point Likert scale) were used to evaluate nutrition. While the total scores of these items ranged between 16 and 64, scores < 33.3%, 33.3% - 66.6%, and > 66.6% of the maximum scores were considered to represent poor, fairly good, and proper nutrition, respectively (14). The 27-item long-form International Physical Activity Questionnaire (IPAQ), whose validity and reliability had been previously confirmed by numerous studies was administered to measure physical activity (16, 17). The IPAQ calculates the energy requirement of each activity by multiplying its metabolic equivalent of task (MET) by the minutes performed (MET-minute) per week. After summing all energy requirements, values below 600, 600-3000, and over 3000 MET-minute/week were considered to show low, moderate, and high levels of physical activity, respectively. In order that all of life sub groups Can be calculated together, physical activity was scored on a seven-point Likert scale from 1 to seven indicating 0 - 499, 500 - 999, 1000 - 1499, 1500 - 1999, 2000 - 2499, 2500 - 2999, and 3000 + MET - minute/week, respectively.

A 12-item valid and reliable scale was employed to evaluate perceived social support in three domains of family, friends, and significant others (18). Since each item was scored 1-7, the total scores ranged between 12 and 84. Low, moderate, and high levels of perceived social support were indicated by scores 12 - 48, 49 - 68, and higher than 68, respectively. Moreover, the responsibility for health was examined through seven researcher - made items based on a four-point Likert scale (7 < total score < 28). Scores <33.3%, 33.3% - 66.6%, and > 66.6% of the maximum scores corresponded to low, moderate, and high responsibility for health. In Iran Sararoudi *et al.* (2011) reported Cronbach's alpha 0.84 for the scale and 0.90, 0.93 and 0.85, respectively for friends, significant others and family subscale. (19) Mirabzadeh *et al* (2013) reported Cronbach's alpha coefficient and the intra-class correlation coefficient (ICC) for this tool 0.89 and 0.92, respectively (20).

The participants’ cigarette and opiate smoking habits were studied using seven researcher-designed items with a total score of 18. Scores lower than 6, 6-12, and higher than 12 represented High, Moderate, and Low level of health behaviors, respectively. Finally, the scores of the five dimensions of lifestyle were calculated as percent values and summed up. Total scores less than 33.3%, 33.3%-66.6%, and greater than 66.6% of the total obtainable score were interpreted as unhealthy, relatively healthy, and healthy lifestyle, respectively.

In order to measure the content validity of the lifestyle questionnaire, it was distributed among 10 faculty members of the School of Nursing and Midwifery (Shahid Beheshti University of Medical Sciences, Tehran, Iran) and two nutritionists. They were requested to carefully read all items and provide their comments. The test-retest reliability of the questionnaire was also assessed (External consistency). Pearson’s correlation coefficient and Cronbach’s alpha) Internal consistency( for the lifestyle questionnaire and its dimensions were computed as 0.7, which was within the acceptable range. In the dimensions of nutrition, responsibility for health, and inappropriate health, the Pearson’s correlation coefficient were 0.97 - 0.97 - 0.98, respectively. 

Cronbach's alpha coefficients in all aspects of nutrition, responsibility for health, inappropriate health were 0.76 - 0.76 - 0.75, respectively The study was approved by the ethic committee of Shahid Beheshti University of Medical Sciences, Tehran, Iran (SBMU.REC.1392. 285). 

**Statistical analysis**

The collected data were presented using descriptive indices such as mean, standard deviation for quantitative data, frequency and percentage for qualitative data. Chi-square, t tests, GEE (Generalized Estimating equation) test, and logistic regression analysis were performed to analyze the data. The P values less than 0.05 were considered significant in all analyses.

## Results

Fertile and infertile women as well as fertile and infertile men were significantly different in terms of age (P= 0.04), ethnicity (p< 0.001), and education level (P= 0.006). Furthermore, there was a significant difference between fertile and infertile females in occupation (p< 0.001), and between fertile and infertile males in occupational exposure to substances (P= 0.002) (Table I).

Most fertile women (62%) had gotten pregnant only once. The duration of infertility in 70.4% of the infertile couples was 36 months and ovulatory disorders were responsible for 40.7% of cases of infertility. The two groups had no significant differences in family size ،age،or type of ownership.

According to the scores of the lifestyle questionnaire, fertile and infertile women were significantly different in terms of nutrition (p< 0.001), physical activity (p= 0.03). Similarly, fertile and infertile men had significant differences in inappropriate health behaviors, responsibility for health, physical activity, and perceived social support (p< 0.001) (Table II). 

Moreover, significant differences in all dimensions of lifestyle, except nutrition, were detected between fertile and infertile couples (004) (Table III).

**Table I:** Demographic and fertility characteristics comparison of fertile and infertile men and women

**Variables**	**Infertile men**	**Fertile men**	**p-value**	**Infertile women**	**Fertile women**	**p-value**
Age (year) (Mean±SD)	32.30±4.62	30.40±5.41	0.040*	30.43±5.05	28.91±5.50	0.040*
**Ethnicity %** **(n)**						
Kord	71.3 (77)	75 (81)	0.020**	88 (95)	66.7 (72)	0.000**
Fars	13.9 (15)	24.1 (26)		4.6 (5)	33.3 (36)	
Lur	14.8 (16)	0.9 (1)		7.4 (8)	0	
**Education level %(n)**						
High school and lower	35.7 (58)	66.7 (72)	0.004**	69.4 (75)	66.7 (72)	0.006**
Collegiate	64.3 (50)	33.3 (36)		30.6 (33)	33.3 (36)	
**Occupation**						
House wife	-	-		71.3 (77)	69.4 (75)	0.000**
Worker	37 (40)	31.5 (34)	0.480**	(2)	2.8 (3)	
Employee	19.4 (21)	32.4 (35)		5.6 (6)	13.9 (15)	
Other	43.6 (47)	36.1 (39)		21.3 (23)	13.9 (15)	
**Occupational Exposure %(n)**						
Waste home	7.4 (8)	9.3 (10)		63.8 (69)	73 (79)	
Chemical	13.9 (15)	10.2 (11)	0.002**	1.9 (2)	3.7 (4)	0.220**
Industrial	4.6 (5)	3.6 (4)		3.7 (4)	0	
Paper	28.7 (31)	41.7 (45)		18.5 (20)	16.7 (18)	
Other	45 (49)	35.2 (38)		12.1 (13)	6.6 (7)	

*Student’s *t *test; ** chi-square test

**Table II:** Frequency distribution of scores of lifestyle and its comparison of dimensions among fertile and infertile men and women

**Variables %(n)**	**Infertile men**	**Fertile men**	**p-value**	**Infertile women**	**Fertile women**	**p-value**
**Nutrition**						
Proper	50.9 (55)	40.7 (44)	0.200	48.1 (52)	70.4 (76)	0.001
Fairly good	49.1 (53)	59.3 (64)		51.9 (56)	29.6 (32)	
**Physical activity**						
High	2.8 (3)	0	0.001	20.4 (22)	9.3 (10)	0.030
Moderate	96.3 (104)	87 (94)		73.1 (79)	86.1 (93)	
Low	0.9 (1)	13 (14)		6.5 (7)	4.6 (5)	
**Un** **appropriate health **						
Low	88 (95)	98.1 (106)	0.030	95.4 (103)	98.1 (106)	0.240
Moderate	12 (13)	1.9 (2)		4.6 (5)	1.9 (2)	
**Perceived social support**						
High	16.7 (18)	4.6 (5)	0.001	0	35.2 (38)	0.001
Moderate	83.3 (90)	92.6 (100)		13.9 (15)	59.3 (64)	
Low	0	2.8 (3)		86.1 (93)	5.5 (6)	
**Responsibility for health**						
High	35.2 (38)	76.9 (83)	0.001	75 (81)	77.8 (84)	0.760
Moderate	64.8 (70)	23.1 (25)		25 (27)	22.2 (24)	
**Total life style**						
Healthy	72.2 (78)	81.5 (88)	0.100	69.4 (75)	83.3 (90)	0.030
Relatively healthy	27.8 (30)	88.5 (20)		30.6 (33)	16.7 (18)	

chi-square test

**Table III:** The marginal model for comparison (GEE) of lifestyle and its dimensions between fertile and infertile men and women

**p-value**	**Standard error**	**B** ^^	**Exp (B** ^ **)=OR**	**Lifestyle dimension**	
0.130	0.19	-0.28	0.74	Fertile couples	Nutrition
				Infertile couples	
<0.001	0.31	-1.16	0.31	Fertile couples	Physical activity
				Infertile couples	
<0.001	0.36	-3.13	0.04	Fertile couples	Perceived social support
				Infertile couples	
<0.001	0.19	-1.02	0.37	Fertile couples	Responsibility for health
				Infertile couples	
0.010	0.57	-1.37	0.26	Fertile couples	Un appropriate health behaviors
				Infertile couples	
0.004	0.22	-0.65	0.53	Fertile couples	Total life style
				Infertile couples	

GEE: Generalized estimating equation; Exp: Exponential; OR:Oods Ratio;

^ Standard Beta;

^^ Un standard Beta;

٭ Refrence category

## Discussion

Based on the finding, most studied fertile and infertile couples had appropriate lifestyle. However, there were statistically significant differences between the two groups in terms of physical activity, perceived social support, responsibility for health, and inappropriat health behaviors.

Researches on the relation between lifestyle and fertility has yielded contradicting results. Revonta *et al*. stated that infertile women and fertile men had more favorable nutritional status compared to fertile women and infertile men (28). On the other hand, cigarette smoking and alcohol use were more common in infertile women and fertile men than in the rest of the study population. However, no significant differences in physical activity were observed between fertile and infertile women or fertile and infertile men (13). Homan *et al*. examined various dimensions of lifestyle including nutrition, physical activity, cigarette smoking, alcohol and drug abuse, and caffeine consumption. They found that All couples had unhealthy life style (21). Such inconsistency between the results of various studies can be attributed to the evaluation of different dimensions of lifestyle or the concept as a whole, the administration of different questionnaires, different sample sizes, and unalike cultural and religious contexts. Oborna *et al*. reported the levels of unnatural and harmful fatty acids to be significantly higher in infertile men’s plasma and seminal fluid than in fertile men’s (22). Moreover, Colombo *et al*. calculated proteins, fats, and carbohydrates to comprise 16%, 33%, and 52% of infertile women’s diet, respectively. These women were also found to consume too little high-fiber foods such as fruit and vegetables (23). The difference between these findings and the results of the present research can be justified by the fact that the two groups in previous studies generally matched in terms of age, education, and place of residence. On the other hand, while we applied a researcher-made questionnaire, Oborna *et al*. and Colomba *et al*. respectively used the international Food Frequency questionnaire and laboratory tests to collect data (22, 23). As social and external factors, e.g. education level can increase people’s knowledge about health-related behaviors and their necessity, the favorable nutritional status of infertile men against fertile men in this study might have been caused by their higher levels of education. In addition, the significantly different ethnicity of fertile and infertile women might have contributed to their dissimilar nutritional status (22, 23). 

While the results of this study regarding physical activity were similar to the findings of Homan *et al*. They were not in agreement with the findings of Gudmundsdottir *et al*. (2009), Esmaeilzadeh *et al*. and Wise *et al*. (8, 24-26). Gudmundsdottir *et al*. found fertile women to be less active than infertile women. They reported these women had a high level of physical activity (24). Likewise, most infertile subjects in studies performed by Esmaeilzadeh *et al*. and Wise *et al*. had high levels of physical activity (25, 26). Meanwhile, Wise *et al*. indicated that only 14% of their participants had high levels of physical activity. Regular physical activity has been proved to control blood glucose and insulin levels, adjust luteinizing and follicle-stimulating hormones, and increase the level of testosterone (12). The observed inconsistencies between the results of various studies might have been caused by the use of different questionnaires. Moreover, the participants' perception of intense and moderate physical activity might have affected the obtained results. Therefore, further studies are required to assess the correlations between body mass index and intensity and duration of physical activity in fertile and infertile men and women.

The findings of this study about inappropriat health behaviors did not match the results of previous studies. Ariyanpour et al. failed to establish a significant difference in smoking between fertile couples and the rest of the population (27). However, Braga et al. found 53/2% of infertile men smocked cigarette regularly (14). Wright *et al*. stated that only 9.3% of the participating infertile women were smokers at the time of the study (28). Revonta *et al*. suggested a lack of a significant difference in smoking and alcohol use between fertile and infertile men (13). Nevertheless, nicotine, existing in cigarettes and other tobacco products, has been proved to exert negative effects on ovarian follicles, cell membrane proteins, and genetic content of the sperm through the production of oxygen free radicals, including hydrogen peroxide (25). Meanwhile, considering the specific cultural and religious context in Iran, the study participants, especially women, might have refused to provide accurate data about their smoking habits and other inappropriate health behaviors. Such a fear of stigmatization could have been responsible for low frequency of inappropriate health behaviors in the studied population. On the other hand, the dependence of inappropriate health behaviors on factors such as gender differences, level of stress, depression, and low perceived social support can justify their higher frequency in infertile couples than in fertile participants (29).

Infertile women in the current research had significantly lower perceived social support compared to the other participants. While similar results were published by Wongpakaran et al. and slade *et al*. (30, 18). Studies by Heidari *et al*. in Mashhad (Iran) and Lund et al. in Denmark revealed that most infertile individuals received greater social and emotional support from their families and spouses (31, 32). Interpersonal relationships could prevent impaired secretion of gonadotropins,local effects of catecholamines, and immunological disturbances, which might occur due to the stress caused by infertility (33). The low level of social support in infertile women might be due to the negative feeling of being isolated and different from others and, consequently, the reduction of infertility stress and failure to receive others’ social support, which needs broader studies and consultative and supportive efforts (30).

Similar to the findings of the current study, in a study which was performed by Mercer et al. found higher responsibility for health in women than in men (34). Meanwhile, the contrasting results reported by Nekuei et al. in Isfahan could have probably been caused by improper evaluation of too many risk factors of infertility (35). Finally, when responsibility for health is concerned, acquiring health-related information from sources such as other people and mass media depends on individuals’ mental and psychological characteristics rather than their education level. On the other hand, gender is a determinant of lifestyle-related behaviors that can determine responsibility for health in fertile and infertile women. Disapproval of some of the questions in the area of health behavior was led to their elimination.

## Conclusion

Fertile and infertile couples in the current study were significantly different in terms of physical activity, perceived social support, responsibility for health, and inappropriate health behaviors. Further and broader studies are warranted to facilitate the planning of relevant interventions.

## Note

***This article extracted from M.Sc. thesis. (Tahereh Khosrorad)***

## References

[1] Gaur DS, Talekar MS, Pathak V (2010). Alcohol intake and cigarette smoking: impact of two major lifestyle factors on male fertility. Indian J Pathol Microbiol.

[2] Fraser M, Cooper MA (2010). Myles text book for midwives.

[3] Amin U, Khan N, Ali Bhat I (2015). Prevalence and causes of infertility among women of Jammu and Kashmir. Int J Dev Res.

[4] Gurunath S, Pandian Z, Anderson RA, Bhattacharya S (2011). Defining infertility-a systematic review of prevalence studies. Hum Reprod Update.

[5] Novak E, Berek JS (2012). Berek & Novak's gynecology.

[6] Kamali Fard M, Alizadeh R, Sehati F, Golzadeh M (2007). The effect of lifestyle on the rate af preterm rate. J Ardabil Univ Med Sci.

[7] Griffiths A, Dyer SM, Lord SJ, Pardy C, Fraser IS, Eckermann S (2010). A cost-effectiveness analysis of in-vitro fertilization by maternal age and number of treatment attempts. Hum Reprod.

[8] Homan GF, Davies M, Norman R (2007). The impact of lifestyle factors on reproductive performance in the general population and those undergoing infertility treatment: a review. Hum Reprod Update.

[9] Craft-Rosenberg M, Pehler S-R (2011). Encyclopedia of family health.

[10] Moerbeek HHS, Niehof A (2007). Changing families and their lifestyles.

[11] Thompson C, Mcdonald S (2005). Women's health.

[12] Redman LM (2006). Physical activity and its effects on reproduction. Reprod Biomed Online.

[13] Revonta M, Raitanen J, Sihvo S, Koponen P, Klemetti R, Männistö S (2010). Health and life style among infertile men and women. Sex Reprod Healthc.

[14] Braga DP, Halpern G, Figueira Rde S, Setti AS, Iaconelli A Jr, Borges E Jr (2012). Food intake and social habits in male patients and its relationship to intracytoplasmic sperm injection outcomes. Fertil Steril.

[15] Twigt J, Bolhuis M, Steegers E, Hammiche F, van Inzen W, Laven J (2012). The preconception diet is associated with the chance of ongoing pregnancy in women undergoing IVF/ICSI treatment. Hum Reprod.

[16] Kelishadi R, Ardalan G, Gheiratmand R, Gouya MM, Razaghi EM, Delavari A (2007). Association of physical activity and diatary behaviors in relation to the body mass index in national sample of iranian children and adolescents. Bull Word Health Orga.

[17] Kurtze N, Rangul V, Hustvedt B-E (2008). Reliability and validity of the international physical activity questionnaire in the Nord-Trøndelag health study (HUNT) population of men. BMC Med Res Methodol.

[18] Wongpakaran T, Wongpakaran N, Ruktrakul R (2011). Reliability and validity of the Multidimensional scale of perceived social support (MSPSS): Thai Version. Clin Pract Epidemio Ment Health.

[19] Sararoudi RB, Sanei H, Baghbanian A (2011). The relationship between type D personality and perceived social support in myocardial infarction patients. J Isfahan Univ Med Sci.

[20] Mirabzadeh A, Dolatian M, Forouzan AS, Sajjadi H, Majd HA, Mahmoodi Z (2013). Path analysis associations between perceived social support, stressful life events and other psychosocial risk factors during pregnancy and preterm delivery. Iranian Red Crescent Med J.

[21] Homan G, Litt J, Norman RJ (2012). The FAST study: Fertility ASsessment and advice Targeting lifestyle choices and behaviours: a pilot study. Hum Reprod.

[22] Oborna I, Wojewodka G, De Sanctis JB, Fingerova H, Svobodova M, Brezinova J (2010). Increased lipid peroxidation andabnormal fatty acid profiles in seminal and blood plasma of normozoospermic males from infertile couples. Hum Reprod.

[23] Colombo O, Pinelli G, Comelli M, Marchetti P, Sieri S, Brighenti F (2009). Dietary intakes in infertile women a pilot study. Nutr J.

[24] Gudmundsdottir SL, Flanders WD, Augestad LB (2009). Physical activity and fertility in women: the North-Trøndelag Health Study. Hum Reprod.

[25] Esmaeilzadeh S, Delavar MA, Basirat Z, Shafi H (2012). Physical activity and body mass index among women who have experienced infertility. Arch Med Sci.

[26] Wise LA, Cramer DW, Hornstein MD, Ashby RK, Missmer SA (2011). Physical activity and semen quality among men attending an infertility clinic. Fertil Steril.

[27] Aryanpur M, Tarahomi M, Sharifi H, Heydari G, Hessami Z, Akhoundi M (2011). Comparison of spermatozoa quality in male smokers and nonsmokers of Iranian infertile couples. Int J Fertil Steril.

[28] Wright KP, Trimarchi JR, Allsworth J, Keefe D (2006). The effect of female tobacco smoking on IVF outcomes. Hum Reprod.

[29] Anderheim L, Holter C, Bergh C, Möller A (2006). Does psychological stress affect the outcome of in vitro fertilization?. Hum Reprod.

[30] Slade P, O’Neill C, Simpson AJ, Lashen H (2007). The relationship between perceived stigma, disclosurepatterns, support and distress in new attendees at an infertility clinic. Hum Reprod.

[31] Heidari P, Latifnejad R (2010). Relationship between psychosocial factors and marital satisfaction in infertile women. J Qazvin Univ Med Sci.

[32] Lund R, Sejbaek CS, Christensen U, Schmidt L (2009). The impact of social relations on the incidence of severe depressive symptoms among infertile women and men. Hum Reprod.

[33] Hashemi S, Simbar M, Ramezani-Tehrani F, Shams J, Majd HA (2012). Anxiety and success of in vitro fertilization. Europ J Obstet Gynecol Reprod Biol.

[34] Mercer CH, Fenton KA, Johnson AM, Wellings K, Macdowall W, McManus S (2003). Sexual function problems and help seeking behaviour in Britain: national probability sample survey. BMJ.

[35] Nekuei N, Kazemi A, Ehsanpur S, Beigi NMA (2013). Preconception risk assessment of infertile couples. Iran J Nurs Midwifery Res.

